# Consumption of commercial and traditional sugar-sweetened beverages among adolescents in Pakistan: evidence from a national survey

**DOI:** 10.3389/fnut.2025.1679917

**Published:** 2025-11-26

**Authors:** Saima Afaq, Damith Chandrasenage, Urooj Ashfaq, Midhat Farzeen, Romaina Iqbal, Marc Suhrcke, Kamran Siddiqi, Mona Kanaan, Gerardo A. Zavala

**Affiliations:** 1 Department of Health Sciences, University of York, York, United Kingdom; 2 Khyber Medical University (KMU), Institute of Public Health & Social Sciences (IPH&SS), Peshawar, Pakistan; 3 Department of Community Health Sciences, Agha Khan University, Karachi, Pakistan; 4 Luxembourg Institute of Socio-Economic Research, Esch-sur-Alzette, Luxembourg

**Keywords:** sugar-sweeten beverages, adolescents, Pakistan, LMIC (low- and middle-income countries), non-communicable diseases, commercial beverages, traditional beverages, out-of-school adolescent

## Abstract

**Background:**

Pakistan faces a growing burden of adolescent overweight, early-onset diabetes, and one of the world’s highest adult diabetes prevalence rates. Yet, nationally representative data on adolescents’ sugar-sweetened beverages (SSBs) consumption are lacking. This study addresses this evidence gap by examining consumption patterns and sociodemographic determinants of SSB intake, including both commercial (packaged) and traditional (home-prepared) drinks, among 10–16-year-olds in Pakistan.

**Methods:**

We analysed data from the nationally representative TAP (Tobacco & Adolescent Health in Pakistan) survey, conducted from December 2023 to May 2024, including 14,232 adolescents (63% in-school, 37% out-of-school) from nine districts. Weekly frequency of two SSB categories (‘commercial’ (packaged) including soft drinks, fruit drinks, energy drinks; and ‘traditional’ including traditional sweetened home-prepared beverages) was assessed. Consumption was categorised as low, moderate, or high. Weighted descriptive statistics and proportional/generalised ordinal logistic regression were used to examine associations with sociodemographic variables.

**Results:**

Overall, 70.5% of adolescents reported high total SSB intake (>7 times per week). High consumption was reported in 17.3% for commercial SSBs and 38.2% for traditional SSBs. In adjusted models, males (OR = 1.32, 95% CI: 1.16–1.51), older adolescents (OR = 1.39, 95% CI: 1.20–1.61), and out-of-school youth (OR = 1.48, 95% CI: 1.26–1.74) had greater odds of high total intake, while higher female caregiver’s education was protective (OR = 0.68, 95% CI: 0.54–0.86). Patterns for commercial SSBs were broadly similar, though male caregiver’s education was positively associated. Traditional SSBs also followed these trends, with stronger associations for out-of-school adolescents (OR = 2.05, 95% CI: 1.77–2.37) and rural residence (OR = 1.14, 95% CI: 1.01–1.28).

**Conclusion:**

SSB consumption including both commercial and traditional beverages is widespread among adolescents in Pakistan. Intake patterns vary significantly by sex, schooling, caregiver education and urbanicity. Policies should prioritise both school and community interventions, implement fiscal and labelling policies, and engage caregivers. Future research should assess portion sizes, nutrient profiles, and strategies to shift social norms around sugar use in beverages.

## Introduction

Sugar-sweetened beverages (SSBs), i.e., beverages that have added sugar, are a major source of calories in diets globally (1, 2). Between 1990 and 2018, global intake among children and adolescents aged 3–19 years increased by 23%, (3) with particularly rapid growth in low- and middle-income countries (LMICs) driven by urbanisation and aggressive marketing strategies, which are particularly aimed at young populations (1, 4, 5).

This rise contributes substantially to the burden of diet-related non-communicable diseases (NCDs), which are now a leading cause of morbidity and mortality worldwide. Globally, in 2020, SSB consumption was estimated to account for 2.2 million cases of type 2 diabetes, 1.2 million cases of cardiovascular disease, and 340,000 related deaths (6). Between 1990 and 2019, global non-communicable diseases (NCDs) related deaths attributable to high SSB intake rose from ~150,000 to 242,000, with Disability-Adjusted Life Years (DALYs) increasing from ~ 3.7 million to over 6 million (7). Among children and adolescents, SSBs remain a leading source of excess dietary sugar and are associated with obesity, type 2 diabetes, cardiovascular disease, and premature mortality (8–11) as well as poorer academic performance, dental caries, and more sedentary behaviors (12).

This rising SSB consumption is especially concerning in LMICs (13, 14), particularly in high NCD burden countries, such as Pakistan (15). Pakistan ranks tenth globally for childhood obesity prevalence with an estimated 9.5% of children under five affected in 2018 (9.7% among boys and 9.2% among girls) (16). Projections suggest that 5.4 million school-aged children in the country could be obese by 2030 (17). The situation is compounded by a high burden of youth-onset type 2 diabetes, with an incidence rate of 88 per 100,000 among children and adolescents in 2021 (18). Such early onset is likely to increase adult disease burden, with national diabetes prevalence already reaching 31.4% among adults in 2024, among the highest globally (19). Given these immediate and long-term health risks, reducing SSB consumption in children and adolescents is a priority globally. In its 2015 guidelines, the World Health Organisation (WHO) recommends limiting free sugar (including sugars from SSBs) intake to less than 10% of daily energy, and ideally below 5% (6).

To support effective strategies for reducing SSB consumption, it is essential to understand consumption patterns, determinants, and disparities across population subgroups (7). Global analyses such as the Global Dietary Database (2018) and recent work by (3) highlight broad disparities by age, urbanity, and parental education, with adolescents aged 13–18 years consistently consuming more SSBs than younger children. However, for Pakistan, these estimates are primarily drawn from the 2009 Global School-based Student Health Survey (GSHS), which, while nationally representative of school-going adolescents, is now 16 years old and includes only those aged 13–15 years. It therefore excludes both younger and older adolescents, and does not capture traditional home prepared sweetened beverages (3). Moreover, most international and national surveys including the Global Youth Tobacco Survey (GYTS) and the GSHS often lack subnational and socio-demographic disaggregation (20) focusing exclusively on school-going adolescents, overlooking out-of-school youth, who represent approximately 23% of adolescents aged 10–16 years in Pakistan (21). In addition, existing global datasets define SSBs narrowly, focusing on commercially packaged beverages and ignoring widely consumed traditional home-prepared drinks such as chai (tea with sugar and milk), sweetened milk, and lassi. These omissions risk underestimating total SSB consumption in cultural contexts like Pakistan, where traditional beverages are a major source of added sugar (22).

This study addresses these evidence gaps by providing nationally representative estimates of total, commercial (packaged), and traditional (home-prepared) SSB consumption among both in-school and out-of-school adolescents in Pakistan. By providing a more granular and context-specific understanding of adolescent SSB intake, this research aims to inform targeted, culturally relevant policies to reduce SSB consumption and its contribution to the rising burden of non-communicable diseases in Pakistan. This is a timely contribution, as the policy interest in SSB-related policy activities is increasing in the country, as exemplified by bans on sugary drinks in school canteens in Sindh and pending legislation in Punjab (23, 24).

## Methods

### Data source

We conducted a secondary data analysis of the TAP (Tobacco Control Policies and the Health of Adolescents in Pakistan) cross-sectional survey conducted between December 2023 and May 2024. The primary aim of the survey was to explore current use, exposure, access and susceptibility toward tobacco and nicotine products among 10–16 year old adolescents that are in school or out-of-school. In addition to tobacco-related indicators, TAP also collected data on adolescents’ SSB consumption, including both commercial (packaged) and traditional (home prepared) SSBs and a wide range of demographic and socio-economic variables as part of the survey.

### Sampling and participants

The TAP survey employed a multi-stage stratified random sampling technique to recruit a nationally representative sample of in-school and out-of-school adolescents across nine districts in Pakistan, with two districts each from four provinces Punjab, Sindh, Khyber Pakhtunkhwa (KPK), and Balochistan and the federal capital, Islamabad. Districts were selected using maximum variation sampling based on out-of-school adolescent rates reported in the Pakistan Social and Living Standards Measurement (PSLM) Survey 2019–2020 (25). Districts represent administrative units within Pakistan’s four provinces and capital territory. Further details of study design, sampling procedures etc. are available in TAP survey paper (26).

For the school-based component, 9,011 adolescents were recruited from 180 secondary schools using a three-stage proportional stratified random sampling design. First, 90 administrative units (Patwar circles) were selected with probability proportional to size (PPS), ensuring proportional coverage of rural and urban areas. Second, two schools were randomly selected per circle, stratified by gender where possible. Third, 50 students were randomly selected from grades 6 to 10, ensuring coverage of ages 10–16 and gender balance.

For the out-of-school component, 5,221 adolescents were recruited through a parallel three-stage approach across 72 enumeration blocks. Household mapping was conducted in each enumeration block to identify eligible adolescents, and 60 households per block were randomly selected. Where insufficient eligible households were identified, additional enumeration blocks were selected within the same circle. If the required sample could not be achieved through household listing, additional recruitment was done through local organisations and social mobilizers for identifying children in community settings (markets they worked, bus stops etc.)

Eligible participants were adolescents aged 10–16 years, either enrolled in school (grades 6–10) or identified as out-of-school through household mapping. Children outside this age range or those without parental consent/adolescent assent were excluded.

The sample size for the TAP survey was calculated using the probability proportional to size (PPS) method; therefore, the frequency of adolescents recruited from each province reflects the population of the province. Assumptions for sample size calculation included: an intraclass correlation coefficient (ICC) of 0.2, a tobacco susceptibility of 10%, (27) a 4% margin of error, and an anticipated 85% response rate (28), accounting for cluster design and subgroup analyses. This yielded target samples of approximately 9,000 in-school and 4,320 out-of-school adolescents. Sampling weights were calculated for both components (school and out-of-school) to account for design effects (24). The detailed procedure for weight calculation is provided in Supplementary File 1.

Parental consent (opt-out) and child assent (opt-in) were obtained before participation.

#### Measures

##### SSB consumption

We defined SSBs according to the World Health Organization (WHO) definition of beverages containing “free sugars,” which includes carbonated and non-carbonated soft drinks, fruit and vegetable juices and drinks with added sugar, liquid and powder concentrates, flavoured water, energy and sports drinks, ready-to-drink tea and coffee, and flavoured milk drinks (29). In line with this, our study classified SSBs as carbonated soft drinks, fruit drinks with added sugar, energy drinks, and traditional home-prepared drinks such as sweetened tea, milk-based, and yogurt-based beverages. Packaged flavoured milk and yogurt drinks were not explicitly captured in the TAP questionnaire, which may lead to some underestimation. 100% fruit juice was excluded because it does not contain added sugars (30).

To estimate SSB, we used a subset of questions that specifically addressed SSB intake, from a food frequency questionnaire (FFQ) validated for adolescents (29). These questions were adapted for cultural relevance through expert review and adolescent pre-testing, then translated into local languages. Adolescents reported their consumption in the past 7 days using six frequency categories, with follow-up questions for daily/multiple daily consumption: (i) Never, (ii) 1 time per week, (iii) 2–4 times per week, (iv) 5–6 times per week, (v) Once a day, every day, and (vi) Every day, more than once. Participants who selected the last option (vi) were asked a follow-up question for daily/multiple daily consumption.

Beverages were classified into two categories: commercial (packaged) and traditional (home prepared). Commercial SSBs included carbonated soft drinks (e.g., Coke, Fanta, Sprite), fruit drinks with added sugar (e.g., Tang, Rooh Afza, Jam e Shireen), and energy drinks. Traditional SSBs referred to home-prepared drinks such as sweetened tea, milk-based and yogurt-based beverages. Packaged flavoured milk and yogurt drinks were not explicitly included in the questionnaire, which may lead to underestimation. We assessed consumption in terms of frequency rather than volume, given the challenges of estimating standard serving sizes for traditional SSBs and the cognitive burden of volume recall among adolescents (29). This approach aligns with global adolescent dietary surveys (3) and allows for comparability.

The primary outcomes of this study were (i) total SSB consumption, defined as the combined weekly frequency of all four beverage categories and (ii) commercial SSB consumption (iii) traditional SSB consumption. Weekly frequency was estimated by assigning midpoint values to categorical responses: Never = 0; 1 per week = 1; 2–4 per week = 3; 5–6 per week = 5.5; once daily = 7. For participants reporting more than once per day, weekly totals were based on the reported number; if the exact figure was not recalled, a conservative minimum of 14/week was assigned. This affected 4.4% of commercial and 19% of traditional SSB observations. The scoring method is summarised in Supplementary File 2, Table S1. To enable meaningful analysis while minimising the effects of small subgroup sizes and ensuring alignment with global SSB guidelines and best practices (31), we categorised the outcome variables into ordered levels for regression analysis: low intake (0–1/week), moderate intake (2–7/week), and high intake (>7/week).

##### Covariates

We used data on demographic and socio-economic characteristics: adolescents’ sex (female and male), age group (10–12 years and 13–16 years), school status (in school and out-of-school), place of residence (urban and rural), and geographical region (KPK, Punjab, Sindh, Balochistan and Islamabad). Socio-economic variables included household wealth index (low, middle, high), caregiver education (separate for male and female: no formal education, primary, secondary, higher), and parental employment (none, father only, mother only, both). The wealth index was derived using principal component analysis (PCA) of household asset ownership, including the presence of outdoor space (garden, yard, balcony, veranda), flush toilet, television, refrigerator, car, and moped/scooter/motorcycle. Variables with little discriminatory power—those owned by more than 95% (e.g., electricity, mobile phone) or fewer than 5% (e.g., radio, fixed telephone)—were excluded. The resulting composite index was divided into three categories (low, middle, high) to reflect socioeconomic stratification. See Section 2.2 in the Supplementary file 2 document for details on the calculation.

## Statistical analysis

Descriptive statistics were computed using sampling weights to ensure population representativeness. Weighted frequencies and percentages of adolescents were calculated across SSB consumption categories, stratified by socio-demographic characteristics.

We used a proportional odds logistic regression model (ordinal logistic regression) to assess the association between sociodemographic characteristics and levels of SSB consumption (low, moderate, high). This model accounts for the ordered nature of the outcome and is more efficient than separate binary or multinomial models. It estimates the odds of being in a higher consumption category versus all lower categories, assuming the relationship between predictors and outcome is consistent across thresholds (the proportional odds assumption).

Separate models were fitted for three outcomes: total SSB consumption, Commercial SSB consumption, and Traditional SSB consumption. All models were adjusted for age group, sex, school attendance, parental education, parental occupation, household wealth index, urban/rural residence, and province.

For total SSB consumption, tests indicated that the Ordinal logistic regression was initially applied; however, for the total SSB outcome, tests revealed that the proportional odds assumption was violated for certain predictors, particularly parental occupation and province. Therefore, a generalised ordinal logistic regression model [Partial Proportional Odds (PPO) model] was used, allowing selected covariates to vary across outcome thresholds (32).

All models incorporated sampling weights, and results are presented as adjusted odds ratios (ORs) with 95% confidence intervals and *p*-values. A two-sided *p*-value <0.05 was considered statistically significant.

This study adheres to the Strengthening the Reporting of Observational Studies in Epidemiology (STROBE) guidelines for cross-sectional studies to ensure transparency and completeness in reporting (33). All analyses were conducted using Stata version 18.5-StataNow, (Stata Corp, 2024). The statistical code used for the analyses will be available on GitHub upon publication, and the dataset used in this study is available upon reasonable request from the corresponding author.

### Ethical considerations

Ethical approval for the original survey was obtained from the Health Sciences Research Governance Committee, Department of Health Sciences, University of York (UK) (HSRGC/2023/566/D), and the National Bioethics Committee (Pakistan) (Reference No. NBC-973), and all data collection followed standard ethical protocols. Parental opt-out consent and adolescent assent were secured before participation in the original survey. For this secondary data analysis, de-identified data was used to ensure confidentiality and anonymity.

## Results

### Characteristics of participants

In the TAP study, a total of 14,232 adolescents were surveyed, of whom 64% (n = 9,106) were boys. Thirty percent were aged 10–12 years, while 70% were aged 13–15 years (Table 1). The sample included 9,011 school-going (63%) and 5,221 out-of-school adolescents (37%). The majority of adolescents (80.6%, *n* = 11,469) reported that only their fathers were employed, followed by both parents working (12.3%, *n* = 1750) and only mothers employed (3.8%, *n* = 538). The household wealth distribution was relatively even: 33.9% (*n* = 4,827) of adolescents were from low wealth index households, 34.3% (*n* = 4,874) from middle wealth households, and 31.8% (*n* = 4,531) from high wealth households. Educational attainment was low among caregivers, with 57.3% (*n* = 8,159) of female caregivers and 35.3% (*n* = 5,017) of male caregivers reporting no formal education. Only 9.2% (*n* = 1,314) of female caregivers and 18.0% (*n* = 2,567) of male caregivers had attained higher education. Participants resided in both rural (54.7%, *n* = 7,782) and urban (45.3%, *n* = 6,450) areas. The sample was drawn from five areas: four provinces—Khyber Pakhtunkhwa, Sindh, Balochistan, and the capital Islamabad—with the highest representation from Punjab (35.4%, *n* = 5,035) (Table 1).

**Table 1:** Characteristics of the study sample (total and by sex and school status) among adolescents in Pakistan, 2023 & 2024, *N* = 14,232.

	Female			Male			Overall
	Out-of-school	In-school	Total	Out-of-school	In-school	Total	
Total, *N* (%)	1,678 (100)	3,448 (100)	5,126 (100)	3,543 (100)	5,563 (100)	9,106 (100)	14,232 (100)
Age (Mean, SD)	12.45 (1.91)	13.77 (1.5)	13.34 (1.76)	12.99 (1.91)	13.93 (1.56)	13.57 (1.76)	13.48 (1.77)
Age groups (years), *N* (%)							
10–12	921 (54.9)	744 (21.6)	1,665 (32.5)	1,482 (41.8)	1,113 (20.0)	2,595 (28.5)	4,260 (29.9)
13–16	757 (45.1)	2,704 (78.4)	3,461 (67.5)	2061 (58.2)	4,450 (80.0)	6,511 (71.5)	9,972 (70.1)
Wealth Index, *N* (%)							
Low	935 (55.7)	609 (17.7)	1,544 (30.1)	1794 (50.6)	1,489 (26.8)	3,283 (36.1)	4,827 (33.9)
Middle	515 (30.7)	1,217 (35.3)	1732 (33.8)	1,077 (30.4)	2065 (37.1)	3,142 (34.5)	4,874 (34.3)
High	228 (13.6)	1,622 (47.0)	1850 (36.1)	672 (19.0)	2009 (36.1)	2,681 (29.4)	4,531 (31.8)
Female caregiver education, *N* (%)							
No education	1,340 (79.9)	1,393 (40.4)	2,733 (53.3)	2,638 (74.5)	2,788 (50.1)	5,426 (59.6)	8,159 (57.3)
Primary	206 (12.3)	627 (18.2)	833 (16.3)	403 (11.4)	850 (15.3)	1,253 (13.8)	2086 (14.7)
Secondary	103 (6.1)	808 (23.4)	911 (17.8)	248 (7.0)	1,024 (18.4)	1,272 (14)	2,183 (15.3)
Higher education	14 (0.8)	531 (15.4)	545 (10.6)	72 (2.0)	697 (12.5)	769 (8.4)	1,314 (9.2)
Do not know	15 (0.9)	89 (2.6)	104 (2.0)	182 (5.1)	204 (3.7)	386 (4.2)	490 (3.4)
Male caregiver education, *N* (%)							
No education	947 (56.4)	742 (21.5)	1,689 (33.0)	1787 (50.4)	1,541 (27.7)	3,328 (36.6)	5,017 (35.3)
Primary	294 (17.5)	466 (13.5)	760 (14.8)	711 (20.1)	841 (15.1)	1,552 (17.0)	2,312 (16.3)
Secondary	301 (17.9)	1,222 (35.4)	1,523 (29.7)	646 (18.2)	1,693 (30.4)	2,339 (25.7)	3,862 (27.1)
Higher education	117 (7.0)	902 (26.2)	1,019 (19.9)	233 (6.6)	1,315 (23.6)	1,548 (17.0)	2,567 (18.0)
Do not know	19 (1.1)	116 (3.4)	135 (2.6)	166 (4.7)	173 (3.1)	339 (3.7)	474 (3.3)
Parents’ occupation status, *N* (%)							
None of them	31 (1.9)	65 (1.9)	96 (1.9)	135 (3.8)	145 (2.6)	280 (3.1)	376 (2.6)
Father only	1,235 (73.6)	2,855 (82.8)	4,090 (79.8)	2,781 (78.5)	4,598 (82.7)	7,379 (81)	11,469 (80.6)
Mother only	92 (5.5)	146 (4.2)	238 (4.6)	125 (3.5)	175 (3.2)	300 (3.3)	538 (3.8)
Both	310 (18.5)	366 (10.6)	676 (13.2)	488 (13.8)	586 (10.5)	1,074 (11.8)	1750 (12.3)
Do not know	10 (0.6)	16 (0.5)	26 (0.5)	14 (0.4)	59 (1.1)	73 (0.8)	99 (0.7)
Resident area, *N* (%)							
Urban	514 (30.6)	1,675 (48.6)	2,189 (42.7)	1,631 (46)	2,630 (47.3)	4,261 (46.8)	6,450 (45.3)
Rural	1,164 (69.4)	1773 (51.4)	2,937 (57.3)	1912 (54)	2,933 (52.7)	4,845 (53.2)	7,782 (54.7)
Province, *N* (%)							
KPK	232 (13.8)	549 (15.9)	781 (15.2)	661 (18.7)	1,051 (18.9)	1712 (18.8)	2,493 (17.5)
Punjab	633 (37.7)	1,423 (41.3)	2056 (40.1)	1,148 (32.4)	1831 (32.9)	2,979 (32.7)	5,035 (35.4)
Sindh	407 (24.3)	879 (25.5)	1,286 (25.1)	1,022 (28.9)	1725 (31.0)	2,747 (30.2)	4,033 (28.3)
Baluchistan	387 (23.1)	459 (13.3)	846 (16.5)	518 (14.6)	693 (12.5)	1,211 (13.3)	2057 (14.5)
Islamabad	19 (1.1)	138 (4.0)	157 (3.1)	194 (5.5)	263 (4.7)	457 (5.0)	614 (4.3)
Total SSB consumption per week, *N* (%)							
Zero	75 (4.5)	158 (4.6)	233 (4.6)	97 (2.7)	208 (3.7)	305 (3.4)	538 (3.78)
One	75 (4.5)	177 (5.1)	252 (4.9)	59 (1.7)	190 (3.4)	249 (2.7)	501 (3.52)
Two-four	99 (5.9)	211 (6.1)	310 (6.1)	182 (5.1)	417 (7.5)	599 (6.6)	909 (6.39)
Five-six	29 (1.7)	17 (0.5)	46 (0.9)	39 (1.1)	37 (0.7)	76 (0.8)	122 (0.86)
Seven	324 (19.3)	578 (16.8)	902 (17.6)	451 (12.7)	778 (14.0)	1,229 (13.5)	2,131 (14.97)
More than seven	1,076 (64.1)	2,307 (66.9)	3,383 (66.0)	2,715 (76.6)	3,933 (70.7)	6,648 (73.0)	10,031 (70.48)
Commercial SSB consumption per week, *N* (%)							
Zero	764 (45.5)	1,119 (32.5)	1883 (36.7)	1,245 (35.1)	1,629 (29.3)	2,874 (31.6)	4,757 (33.4)
One	265 (15.8)	716 (20.8)	981 (19.1)	552 (15.6)	979 (17.6)	1,531 (16.8)	2,512 (17.7)
Two-four	192 (11.4)	418 (12.1)	610 (11.9)	699 (19.7)	1,049 (18.9)	1748 (19.2)	2,358 (16.6)
Five-six	36 (2.2)	146 (4.2)	182 (3.6)	229 (6.5)	146 (2.6)	375 (4.1)	557 (3.9)
Seven	49 (2.9)	212 (6.2)	261 (5.1)	136 (3.8)	364 (6.5)	500 (5.5)	761 (5.4)
More than seven	372 (22.2)	837 (24.3)	1,209 (23.6)	682 (19.3)	1,396 (25.1)	2078 (22.8)	3,287 (23.1)
Traditional SSB consumption per week, *N* (%)							
Zero	151 (9.0)	336 (9.7)	487 (9.5)	217 (6.1)	484 (8.7)	701 (7.7)	1,188 (8.4)
One	177 (10.6)	442 (12.8)	619 (12.1)	269 (7.6)	719 (12.9)	988 (10.9)	1,607 (11.3)
Two-four	84 (5.0)	124 (3.6)	208 (4.1)	97 (2.7)	154 (2.8)	251 (2.8)	459 (3.2)
Five-six	95 (5.7)	121 (3.5)	216 (4.2)	187 (5.3)	249 (4.5)	436 (4.8)	652 (4.6)
Seven	557 (33.2)	1,406 (40.8)	1963 (38.3)	1,056 (29.8)	2,133 (38.3)	3,189 (35.0)	5,152 (36.2)
More than seven	614 (36.6)	1,019 (29.6)	1,633 (31.9)	1717 (48.5)	1824 (32.8)	3,541 (38.9)	5,174 (36.4)

The frequencies (Ns) in the table are unweighted.

### SSB consumption

#### Total SSB consumption

Overall, 70.5% adolescents had high SSB intake, 22.3% had moderate while only 7.2% reported low intake (Table 2). By subgroup, 72.8% of males and 65.4% of females reported high intake. High intake was reported by 72.0% of out-of-school adolescents and 69.7% of school-going adolescents (Table 2). Among adolescents aged 13–16 years, 72.3% had high intake, compared with 65.7% among those aged 10–12 years. Patterns by wealth and female caregiver education varied, with prevalence ranging from 68.8% in low-wealth households to 73.0% in middle-wealth households, and from 72.8% among those with secondary-educated female caregivers to 59.2% among those with higher-educated female caregivers Substantial regional variation was observed, with prevalence estimates of 88.0% in Balochistan, 79.3% in Khyber Pakhtunkhwa, 76.6% in Sindh, 56.4% in Punjab, and 55.0% in Islamabad. Full descriptive results for all subgroups are provided in Table 2.

**Table 2:** Total sugar-sweetened beverage (SSB) consumption per week among adolescents in Pakistan, 2023 & 2024 by socio-demographic factors, *N* = 14,232.

	Zero or one	Two to seven	More than 7	Total	*p*-value^a^
Total, *N* (%)	1,030 (7.2)	3,175 (22.3)	10,027 (70.5)	14,232 (100)	<0.0001
Sex, *N* (%)					
Female	451 (9.9)	1,127 (24.7)	2,982 (65.4)	4,560 (100)	<0.0001
Male	579 (6.0)	2048 (21.2)	7,046 (72.8)	9,672 (100)	
Age (Mean, SD)	13.28 (1.78)	13.29 (1.8)	13.57 (1.75)	13.48 (1.77)	<0.0001
Age groups (years), *N* (%)					
10–12 years old	405 (10.1)	973 (24.2)	2,639 (65.7)	4,017 (100)	<0.0001
13–16 years old	625 (6.1)	2,202 (21.6)	7,388 (72.3)	10,215 (100)	
School status, *N* (%)					
School-going	681 (7.3)	2,155 (23.1)	6,511 (69.7)	9,348 (100)	<0.0001
Out-of-school	349 (7.1)	1,019 (20.9)	3,516 (72.0)	4,884 (100)	
Wealth Index, *N* (%)					
Low	302 (8.1)	860 (23.1)	2,564 (68.8)	3,726 (100)	<0.0001
Middle	281 (5.9)	998 (21.1)	3,455 (73.0)	4,734 (100)	
High	447 (7.8)	1,317 (22.8)	4,008 (69.4)	5,772 (100)	
Female caregiver education, *N* (%)					
No education	488 (6.9)	1,593 (22.4)	5,024 (70.7)	7,105 (100)	<0.0001
Primary	145 (6.3)	486 (21.1)	1,670 (72.6)	2,301 (100)	
Secondary	170 (6.2)	573 (20.9)	1991 (72.8)	2,734 (100)	
Higher education	188 (12.6)	423 (28.3)	886 (59.2)	1,497 (100)	
Male caregiver education, *N* (%)					
No education	358 (8.8)	994 (24.3)	2,745 (67.0)	4,098 (100)	0.08
Primary	142 (6.0)	545 (22.8)	1,698 (71.2)	2,384 (100)	
Secondary	251 (5.6)	911 (20.5)	3,283 (73.9)	4,445 (100)	
Higher education	237 (8.6)	629 (22.9)	1882 (68.5)	2,747 (100)	
Parents’ occupation status, *N* (%)					
None of them	54 (13.2)	80 (19.4)	279 (67.4)	413 (100)	<0.0001
Father only	810 (6.8)	2,660 (22.3)	8,476 (71.0)	11,946 (100)	
Mother only	66 (14.5)	104 (22.7)	287 (62.8)	457 (100)	
Both	76 (5.7)	313 (23.5)	942 (70.8)	1,331 (100)	
Resident area, *N* (%)					
Urban	454 (6.1)	1,511 (20.2)	5,518 (73.7)	7,484 (100)	0.01
Rural	576 (8.5)	1,663 (24.7)	4,509 (66.8)	6,748 (100)	
Province, *N* (%)					
KPK	134 (4.3)	505 (16.4)	2,445 (79.3)	3,084 (100)	<0.0001
Punjab	653 (12.6)	1,612 (31.1)	2,926 (56.4)	5,192 (100)	
Sindh	215 (4.2)	980 (19.2)	3,908 (76.6)	5,103 (100)	
Baluchistan	24 (3.0)	72 (9.0)	738 (88.0)	834 (100)	
Islamabad	4 (18.0)	5 (27.0)	11 (55.0)	20 (100)	

a *p*-values from *χ*^2^ tests for categorical variables and ANOVA for age.

The frequencies (Ns) in the table have been weighted using sampling weights. “Do not know” responses for female caregiver’s education, male caregiver’s education, and parents’ occupation status were excluded; hence, the total sample size in these variables differs from 14,232.

After adjusting for all sociodemographic characteristics (Table 3), several factors were associated with greater odds of consuming moderate-to-high versus low total SSBs; males had higher odds than females (OR = 1.32, 95% CI: 1.16–1.51), adolescents aged 13–16 years had higher odds than those aged 10–12 years (OR = 1.39, 95% CI: 1.20–1.61) and out-of-school adolescents had higher odds than in-school peers (OR = 1.48, 95% CI: 1.26–1.74). Caregiver education showed a mixed pattern: male caregiver secondary education (OR = 1.53, 95% CI: 1.27–1.85) and male caregiver higher education (OR = 1.42, 95% CI: 1.13–1.78) were associated with increased intake, while female caregiver higher education was protective (OR = 0.68, 95% CI: 0.54–0.86). Parental employment status was also significant: adolescents with only the father employed (OR = 2.06, 95% CI: 1.19–3.57) or both parents employed (OR = 2.74, 95% CI: 1.49–5.03) had greater odds of moderate intake compared with those with unemployed parents. Regional variation was pronounced: compared with Khyber Pakhtunkhwa, odds were lower in Punjab (OR = 0.33, 95% CI: 0.27–0.40), Sindh (OR = 0.73, 95% CI: 0.61–0.89), and Islamabad (OR = 0.20, 95% CI: 0.13–0.32), but higher in Balochistan (OR = 1.98, 95% CI: 1.34–2.91).

**Table 3:** Associations between adolescent’s total SSB consumption (number of servings per week) and socio-demographic variables among adolescents in Pakistan, 2023 & 2024, *N* = 13,548.

	Low vs. moderate to high	Moderate vs. high
	OR (95% CI)	OR (95% CI)
Sex		
Female	1.00	1.00
Male	1.32 (1.16, 1.51)	1.32 (1.16, 1.51)
Age groups (years)		
10–12 years old	1.00	1.00
13–16 years old	1.39 (1.20, 1.61)	1.39 (1.20, 1.61)
School status		
School-going	1.00	1.00
Out-of-school	1.48 (1.26, 1.74)	1.48 (1.26, 1.74)
Wealth index, *N* (%)		
Low	1.00	1.00
Middle	1.12 (0.94, 1.35)	1.12 (0.94, 1.35)
High	1.10 (0.92, 1.32)	1.10 (0.92, 1.32)
Female caregiver education		
No education	1.00	1.00
Primary	1.06 (0.88, 1.28)	1.06 (0.88, 1.28)
Secondary	0.98 (0.81, 1.19)	0.98 (0.81, 1.19)
Higher education	0.68 (0.54, 0.86)	0.68 (0.54, 0.86)
Male caregiver education		
No education	1.00	1.00
Primary	1.26 (1.03, 1.55)	1.26 (1.03, 1.55)
Secondary	1.53 (1.27, 1.85)	1.53 (1.27, 1.85)
Higher education	1.42 (1.13, 1.78)	1.42 (1.13, 1.78)
Parents’ occupation status		
None of them	1.00	1.00
Father only	2.06 (1.19, 3.57)	1.30 (0.83, 2.03)
Mother only	0.87 (0.47, 1.60)	0.87 (0.47, 1.60)
Both	2.74 (1.49, 5.03)	1.47 (0.91, 2.37)
Resident area		
Urban	1.00	1.00
Rural	0.89 (0.78, 1.02)	0.89 (0.78, 1.02)
Province		
KPK	1.00	1.00
Punjab	0.33 (0.27, 0.40)	0.33 (0.27, 0.40)
Sindh	0.73 (0.61, 0.89)	0.73 (0.61, 0.89)
Baluchistan	1.98 (1.34, 2.91)	1.98 (1.34, 2.91)
Islamabad	0.20 (0.13, 0.32)	0.32 (0.24, 0.44)

The model included sampling weights.

Each column represents the odds of moving from the lowest category to a higher category.

Patterns were largely consistent for high versus moderate intake. Males, older adolescents, and out-of-school adolescents had significantly greater odds of high intake, while female caregiver higher education remained protective. Regional differences followed the same pattern, with Balochistan showing higher odds and Punjab and Islamabad lower odds compared with Khyber Pakhtunkhwa. Details are provided in Table 3.

#### Commercial SSB consumption

High intake of traditional SSBs was reported by 38.2% of adolescents, while 42.4% had moderate intake and 19.4% reported low intake (Supplementary Table 3). Among subgroups, high intake of traditional SSBs was reported by 44.9% of out-of-school adolescents and 34.7% of in-school adolescents. High intake was 39.1% among rural adolescents and 37.4% among urban adolescents.

In the adjusted model (Table 4), several factors were associated with greater odds of moderate-to-high compared with low commercial SSB intake; males had higher odds than females (OR = 1.35, 95% CI: 1.19–1.52), and adolescents aged 13–16 years had higher odds than those aged 10–12 years (OR = 1.26, 95% CI: 1.09–1.45). Out-of-school adolescents also had higher odds than in-school peers (OR = 1.26, 95% CI: 1.08–1.46). Maternal education showed a graded association: compared with adolescents whose mothers had no formal education, those with mothers who had secondary (OR = 1.40, 95% CI: 1.16–1.67) or higher education (OR = 1.74, 95% CI: 1.40–2.16) had greater odds of intake. Adolescents with employed parents also had higher odds, including those with only the father employed (OR = 1.60, 95% CI: 1.13–2.26), only the mother employed (OR = 1.68, 95% CI: 1.03–2.72), or both parents employed (OR = 1.80, 95% CI: 1.23–2.64) compared with those with unemployed parents. Rural adolescents had lower odds than urban adolescents (OR = 0.76, 95% CI: 0.67–0.86). Strong regional variation was observed: compared with adolescents in Khyber Pakhtunkhwa, odds were higher in Sindh (OR = 1.88, 95% CI: 1.59–2.22) and Balochistan (OR = 11.91, 95% CI: 8.52–16.64), and lower in Punjab (OR = 0.68, 95% CI: 0.57–0.82) and Islamabad (OR = 0.57, 95% CI: 0.43–0.74).

**Table 4:** Associations between commercial SSB consumption (number of servings) per week and socio-demographic variables among adolescents in Pakistan, 2023 & 2024, *N* = 13,548.

	Low vs. moderate to high	Moderate vs. high
	OR (95% CI)	OR (95% CI)
Sex		
Female	1.00	1.00
Male	1.35 (1.19, 1.52)	1.35 (1.19, 1.52)
Age groups (years)		
10–12 years old	1.00	1.00
13–16 years old	1.26 (1.09, 1.45)	1.26 (1.09, 1.45)
School status		
School-going	1.00	1.00
Out-of-School	1.26 (1.08, 1.46)	0.89 (0.73, 1.09)
Wealth Index, *N* (%)		
Low	1.00	1.00
Middle	1.32 (1.11, 1.57)	1.05 (0.85, 1.30)
High	1.27 (1.06, 1.52)	0.98 (0.78, 1.23)
Female caregiver education		
No education	1.00	1.00
Primary	1.07 (0.90, 1.28)	1.07 (0.9, 1.28)
Secondary	1.40 (1.16, 1.67)	1.4 (1.16, 1.67)
Higher education	1.74 (1.40, 2.16)	1.74 (1.40, 2.16)
Male caregiver education		
No education	1.00	1.00
Primary	1.17 (0.97, 1.41)	1.17 (0.97, 1.41)
Secondary	1.08 (0.91, 1.28)	1.08 (0.91, 1.28)
Higher education	1.12 (0.92, 1.36)	1.12 (0.92, 1.36)
Parents’ occupation status		
None of them	1.00	1.00
Father only	1.60 (1.13, 2.26)	1.60 (1.13, 2.26)
Mother only	1.68 (1.03, 2.72)	1.68 (1.03, 2.72)
Both	1.80 (1.23, 2.64)	1.80 (1.23, 2.64)
Resident area		
Urban	1.00	1.00
Rural	0.76 (0.67, 0.86)	0.76 (0.67, 0.86)
Province		
KPK	1.00	1.00
Punjab	0.68 (0.57, 0.82)	0.85 (0.68, 1.06)
Sindh	1.88 (1.59, 2.22)	1.88 (1.59, 2.22)
Baluchistan	11.91 (8.52, 16.64)	28.38 (21.65, 37.21)
Islamabad	0.57 (0.43, 0.74)	0.97 (0.71, 1.31)

The model included sampling weights.

Each column represents the odds of moving from the lowest category to a higher category/category.

For the comparison of high versus moderate intake, patterns were generally consistent. Males, older adolescents, and those with mothers who had secondary or higher education had greater odds of high intake, while rural residence and living in Punjab remained protective. Regional variation was even more pronounced whereas school status was not significantly associated (Table 4).

#### Traditional SSB consumption

High intake of traditional SSBs was reported by 38.2% of adolescents, while 43.5% had moderate intake and 18.3% reported no intake (Supplementary Table 6). Among subgroups, high intake of traditional SSBs was reported by 41.8% of out-of-school adolescents and 36.4% of in-school adolescents. High intake was 39.6% among rural adolescents and 36.7% among urban adolescents. Patterns by sex, age, and caregiver education followed broadly similar trends to commercial SSB consumption; full descriptive estimates with 95% CIs are available in Supplementary Table 6.

In the adjusted model (Table 5), several factors were associated with greater odds of high vs. moderate traditional SSB intake. Males (OR = 1.13, 95% CI: 1.01–1.27), older adolescents aged 13–16 years (OR = 1.60, 95% CI: 1.37–1.86), out-of-school adolescents (OR = 2.05, 95% CI: 1.77–2.37), and rural residents (OR = 1.14, 95% CI: 1.01–1.28) all had higher odds compared with their respective reference groups. Maternal higher education was protective, with lower odds of high vs. low intake (OR = 0.46, 95% CI: 0.36–0.58). Regional variation was marked: compared with Khyber Pakhtunkhwa, odds were lower in Balochistan (OR = 0.28, 95% CI: 0.22–0.35), Punjab (OR = 0.26, 95% CI: 0.22–0.30) and Islamabad (OR = 0.20, 95% CI: 0.15–0.27).

**Table 5:** Associations between traditional SSB consumption (number of servings: zero or one, two to seven and more than seven) per week and socio-demographic variables among adolescents of Pakistan, 2023 & 2024 using generalised ordinal logistic regression model, *N* = 13,548.

	Low vs. moderate to high	Moderate vs. high
	OR (95% CI)	OR (95% CI)
Sex		
Female	1.00	1.00
Male	1.13 (1.01, 1.27)	1.13 (1.01, 1.27)
Age groups (years)		
10–12 years old	1.00	1.00
13–16 years old	1.11 (0.94, 1.31)	1.6 (1.37, 1.86)
School status		
School going	1.00	1.00
Out-of-school	1.05 (0.88, 1.24)	2.05 (1.77, 2.37)
Wealth index		
Low	1.00	1.00
Middle	1.09 (0.94, 1.26)	1.09 (0.94, 1.26)
High	1.02 (0.87, 1.19)	1.02 (0.87, 1.19)
Female caregiver education		
No education	1.00	1.00
Primary	0.93 (0.79, 1.08)	0.93 (0.79, 1.08)
Secondary	0.63 (0.53, 0.74)	0.63 (0.53, 0.74)
Higher education	0.33 (0.26, 0.41)	0.46 (0.36, 0.58)
Male caregiver education		
No education	1.00	1.00
Primary	1.05 (0.88, 1.25)	1.05 (0.88, 1.25)
Secondary	1.5 (1.24, 1.81)	1.21 (1.01, 1.43)
Higher education	1.3 (1.07, 1.57)	1.3 (1.07, 1.57)
Parents’ occupation status		
None of them	1.00	1.00
Father only	1.03 (0.72, 1.47)	1.03 (0.72, 1.47)
Mother only	0.83 (0.49, 1.43)	0.83 (0.49, 1.43)
Both	1.42 (0.95, 2.14)	0.93 (0.63, 1.38)
Resident area		
Urban	1.00	1.00
Rural	1.14 (1.01, 1.28)	1.14 (1.01, 1.28)
Province		
KPD	1.00	1.00
Punjab	0.26 (0.22, 0.30)	0.26 (0.22, 0.30)
Sindh	0.33 (0.27, 0.39)	0.59 (0.50, 0.69)
Baluchistan	0.28 (0.22, 0.35)	0.28 (0.22, 0.35)
Islamabad	0.20 (0.15, 0.27)	0.20 (0.15, 0.27)

The model included sampling weights.

Each column represents the odds of moving from the lowest category to a higher category/category.

## Discussion

This study provides the first nationally representative estimates of SSB consumption among adolescents in Pakistan. Over 70% reported consuming more than seven servings per week, with particularly high intake among males, older adolescents, and out-of-school youth. Urban residence was associated with higher intake of total and commercial, while rural residence was linked to greater consumption of traditional. Female caregiver education was inversely associated with total and traditional SSB intake, whereas male caregiver education showed a positive association. Significant geographic variation was also observed, with Balochistan and Sindh reporting the highest levels of commercial SSB consumption, and adolescents in Punjab and Islamabad reporting the lowest. These provincial findings, however, should be interpreted with caution given wide confidence intervals and the smaller subsamples available for some regions.

Patterns of SSB intake varied by beverage type. Commercial SSBs such as soft drinks and fruit-flavoured beverages were widely consumed, with nearly one in four adolescents reporting high intake. In contrast, traditional SSBs such as sweetened tea, milk, and yogurt-based drinks were even more prevalent, with 38% reporting high weekly intake. Notably, traditional SSBs were more common among males, older adolescents, and rural residents, and patterns by household wealth were less clear.

These findings align with regional trends in South Asia and the Middle East, where adolescents’ SSB intake has increased rapidly (34). A survey of urban adolescents (14–16 years) in Kolkata, India, reported a daily SSB consumption of ~4 servings, amounting to a weekly intake of nearly 28 servings (35). While another study from Jodhpur, India, reported ~6 SSB servings per week among adolescents aged 13–15 years (36). According to the Global Dietary Database, adolescents in Saudi Arabia consume around 9 servings of SSB per week (3). Global modelling estimates from 2018 suggested that Mexico had the highest adolescent SSB consumption worldwide at 10.1 servings per week; however, such estimates typically include only commercially labelled soft drinks (3). Our results suggest that when traditional drinks are included, Pakistani adolescents may exceed many international averages (3, 37).

The increased SSB intake among male and older adolescents reflects global consumption patterns observed in countries such as Australia, the U.S., and China, where these groups consistently report higher intake levels (38–40). These groups may have greater autonomy in food choices, higher exposure to advertising, and lower health literacy (41). The inverse association with maternal education aligns with literature showing that educated mothers are more likely to enforce healthy dietary behaviours (41–44). In contrast, the positive association between paternal education and higher adolescent SSB intake may reflect greater exposure to aspirational consumption norms or permissiveness in adolescents’ dietary choices, rather than household income or occupation, as these were not significant predictors in our models (45). While fathers often contribute financially, maternal education appears to play a more decisive role in shaping household dietary quality in Pakistan (46).

Adolescents from middle-income households were significantly more likely to consume commercial SSBs, but wealth was not a consistent predictor of total or traditional intake. This partial pattern resonates with broader LMIC literature, where middle-income groups are often at the centre of the nutrition transition, exposed to aggressive marketing, shifting toward processed foods, and possessing greater purchasing power than low-income peers but with less dietary restraint or health awareness than higher-income households (47, 48).

Urban adolescents consumed more commercial SSBs, likely due to greater availability and advertising exposure (49), while traditional drinks such as sweetened tea and lassi remained more common in rural areas (50). Despite lower economic development, Balochistan and Sindh showed the highest intake of commercial SSBs, suggesting that regional variations may be shaped by urbanisation, marketing, and cultural norms rather than income alone. Further qualitative and geographic analysis is warranted to unpack these provincial differences (51).

The higher intake among out-of-school adolescents highlights a critical equity issue. Their exclusion from school-based health promotion and limited access to regulated food environments may make them particularly vulnerable (52). Similar findings have been reported in sub-Saharan Africa and Southeast Asia, where out-of-school adolescents have higher exposure to informal and unregulated food sources (53).

### Policy implications and future directions

These findings have significant policy implications. Strengthening school-based nutrition policies across provinces remains essential to restrict adolescents’ access to SSBs, particularly for older adolescents and boys, who reported the highest intake (54). Community-based interventions are equally essential to reach out-of-school adolescents, who remain outside the reach of school health programmes (55). While some provincial authorities have acted, such as the Sindh Food Authority banning sugary drinks in school canteens since 2018, reinforced with renewed directives in 2025, and Karachi schools formally restricting sales of soda and energy drinks (23), implementation has been inconsistent. Similar efforts are emerging in Punjab, where a petition is under consideration to ban sugary beverages in schools (24), and in Islamabad, where the district administration imposed a ban on sales near schools in 2020 (56). However, these interventions are fragmented, often poorly enforced, and do not extend to community-level marketing. Coordinated, province-specific strategies that address both school environments and broader community determinants of SSB consumption are needed. Engaging caregivers, particularly female caregivers, should be a central focus (57).

Globally, front-of-pack nutrition labelling (FOPNL) and health warning labels have been shown to improve consumer understanding and reduce sugary drink purchases by up to 19% (58, 59). Randomised trials in Chile, South Africa, and Mexico have demonstrated that FOPNL policies reduce household SSB intake (60, 61). In Pakistan, however, such labelling remains absent from most beverages, despite being recommended in the Pakistan Dietary Guidelines for Better Nutrition (2018), the WHO Eastern Mediterranean Region Nutrition Strategy (2020–2030), and the WHO Regional Framework for Obesity Prevention (2019–2023) (62–64).

Fiscal measures such as SSB taxation offer a promising intervention. Evidence from Mexico, South Africa, and the U.S. shows that even modest price increases reduce purchases, particularly among heavy consumers (65, 66). The WHO’s 2022 global manual recommends SSB taxation to lower affordability and encourage healthier choices (67). In Pakistan, public support for SSB taxation is high, with 78% of adults supporting such a policy (49). Introducing the SSB tax could generate revenue, reduce healthcare costs, and promote health equity, particularly if revenue is earmarked for health promotion or school nutrition programmes (68, 69). While such a tax would not directly apply to traditional, home-prepared beverages, it could generate revenue for public education campaigns and help reframe sugary drinks, including traditional beverages, as a public health concern.

Culturally sensitive approaches are needed to reduce sugar in traditional home-prepared drinks such as tea, milk, and yogurt-based beverages (70). Public health strategies must also promote water consumption through practical incentives such as installing safe drinking water stations in schools and running behavioral campaigns (71, 72).

### Strengths and limitations

This study provides evidence on the scale and drivers of commercial and traditional SSB consumption among adolescents in Pakistan covering all provinces and the federal capital. The large sample size allowed for stratified analysis across sex, school status, urban–rural residence, and provincial subgroups. Importantly, both in-school and out-of-school adolescents were included, addressing a critical evidence gap as out-of-school youth are typically excluded from international surveys. Another novel strength is the inclusion of traditional, home-prepared SSBs such as sweetened tea, milk, and yogurt-based drinks, which are culturally central but often omitted from global monitoring, leading to more accurate estimates of total SSB intake. Finally, harmonised intake categories and the use of adjusted ordinal regression models, including partial proportional odds where appropriate, improve consistency and robustness of findings. This study also has some limitations. First, its cross-sectional design precludes causal inference. Second, SSB intake was self-reported, making the data subject to recall and social desirability bias. Third, while we measured frequency of consumption, portion sizes and beverage volumes were not recorded. This limits comparability with studies reporting millilitres or grams of sugar intake. However, frequency is a pragmatic proxy, particularly for traditional beverages that lack standard serving sizes. Fourth, although the sample is nationally representative of adolescents aged 10–16, the purposive selection of districts may limit generalisability to all regions. Provincial estimates should also be interpreted with caution due to small subsamples and wide confidence intervals, especially for Balochistan where estimates showed very high odds ratios. Fifth, while we harmonised intake categories across total, commercial, and traditional SSBs (low, moderate, high), these thresholds were pragmatically chosen based on data distribution and may not align with other studies, affecting comparability. Sixth, some beverage types were not explicitly included in the questionnaire, notably commercially available sugar-sweetened milk and yoghurt drinks, which may lead to underestimation. Seventh, we did not adjust for broader potential confounders such as lifestyle factors, health literacy, mental health, or overall dietary intake, as these were not collected in the national survey. Eighth, seasonal variation may have influenced reporting, since data were collected between December and May 2024. Finally, imputation of a conservative minimum (14/week) for approximately one in five traditional SSB consumers means these estimates should be interpreted with caution.

### Conclusion

This study found high levels of SSB intake among adolescents in Pakistan, including both commercially packaged and traditional home prepared beverages. Male adolescents and out-of-school youth were particularly likely to consume SSBs frequently. These findings underscore the need for interventions that are responsive to both commercial and cultural drivers of sugar intake. Policy approaches supported by our findings include targeted school and community-based programmes, particularly for out-of-school adolescents, and education campaigns focused on caregivers. Future research should further examine the volume and nutritional composition of both commercial and traditional drinks to inform comprehensive dietary guidelines and fiscal measures.

## Data Availability

The data analyzed in this study is subject to the following licenses/restrictions: The TAP study dataset is under the control of the project team and has not been publicly released yet. Requests to access these datasets should be directed to saima.afaq@york.ac.uk.
