# Correction: Consumption of commercial and traditional sugar-sweetened beverages among adolescents in Pakistan: evidence from a national survey

**DOI:** 10.3389/fnut.2026.1829233

**Published:** 2026-05-01

**Authors:** Saima Afaq, Damith Chandrasenage, Urooj Ashfaq, Midhat Farzeen, Romaina Iqbal, Marc Suhrcke, Kamran Siddiqi, Mona Kanaan, Gerardo A. Zavala

**Affiliations:** 1 Department of Health Sciences, University of York, York, United Kingdom; 2 Khyber Medical University (KMU), Institute of Public Health & Social Sciences (IPH&SS), Peshawar, Pakistan; 3 Department of Community Health Sciences, Agha Khan University, Karachi, Pakistan; 4 Luxembourg Institute of Socio-Economic Research, Esch-sur-Alzette, Luxembourg

**Keywords:** sugar-sweeten beverages, adolescents, Pakistan, LMIC (low- and middle-income countries), non-communicable diseases, commercial beverages, traditional beverages, out-of-school adolescent

File **Supplementary File 2** has duplicated **Supplementary Tables S1, S2**.. The duplicate tables have been removed, and the **Supplementary material** has been updated.

In the **Abstract**, *Results*, there was an error in the reported percentages. The incorrect sentence read as, “High consumption was reported in 22.3% for commercial SSBs and 38.1% for traditional SSBs.” The correct sentence should read as follows: “High consumption was reported in 17.3% for commercial SSBs and 38.2% for traditional SSBs.”

In **Results**, *Characteristics of participants*, the proportion of adolescents aged 10–12 years and 13–15 years was incorrectly reported. The incorrect sentence read as, “Forty-five percent were aged 10–12 years, while 55% were aged 13–15 years (Table 1)”. The correct sentence should read as follows. “Thirty percent were aged 10–12 years, while 70% were aged 13–15 years (Table 1).”

In **Results**, *SSB Consumption*, paragraph one, there was an error in the 95% confidence intervals presented. The incorrect paragraph read as follows, “Overall, 70.5% (95%CI = 69.7, 71.2) adolescents had high SSB intake, 22.3% (95% CI = 21.6, 23.0) had moderate while only 7.2% (95% CI = 6.8, 7.7) reported low intake (Table 2). By subgroup, 72.8% (95% CI: 71.9–73.7) of males and 65.4% (95% CI: 64.0–66.8) of females reported high intake. High intake was reported by 72.0% (95% CI: 70.7–73.2) of out-of-school adolescents and 69.7% (95% CI: 68.7–70.6) of school-going adolescents (Table 1). Among adolescents aged 13–16 years, 72.3% (95% CI: 71.4–73.2) had high intake, compared with 65.7% (95% CI: 64.2–67.2) among those aged 10–12 years. Patterns by wealth and female caregiver education varied, with prevalence ranging from 68.8% (95% CI: 67.3–70.3) in low-wealth households to 73.0% (95% CI: 71.7–74.2) in middle-wealth households, and from 72.8% (95% CI: 70.7–74.4) among those with secondary-educated female caregivers to 59.2% (95% CI: 56.7–61.6) among those with higher-educated female caregivers Substantial regional variation was observed, with prevalence estimates of 88.0% in Balochistan, 79.3% in Khyber Pakhtunkhwa, 76.6% in Sindh, 56.4% in Punjab, and 55.0% in Islamabad. Full descriptive results for all subgroups are provided in Table 2.” The correct paragraph should read as, “Overall, 70.5% adolescents had high SSB intake, 22.3% had moderate while only 7.2% reported low intake (Table 2). By subgroup, 72.8% of males and 65.4% of females reported high intake. High intake was reported by 72.0% of out-of-school adolescents and 69.7% of school-going adolescents (Table 2). Among adolescents aged 13–16 years, 72.3% had high intake, compared with 65.7% among those aged 10–12 years. Patterns by wealth and female caregiver education varied, with prevalence ranging from 68.8% in low-wealth households to 73.0% in middle-wealth households, and from 72.8% among those with secondary-educated female caregivers to 59.2% among those with higher-educated female caregivers Substantial regional variation was observed, with prevalence estimates of 88.0% in Balochistan, 79.3% in Khyber Pakhtunkhwa, 76.6% in Sindh, 56.4% in Punjab, and 55.0% in Islamabad. Full descriptive results for all subgroups are provided in Table 2.”

In **Results**, *SSB Consumption*, paragraph 4, **Supplementary Table 4** was incorrectly cited. The correct citation should read as “**Supplementary Table 3**”.

In **Results**, *SSB Consumption*, paragraph 4, the reported percentages were incorrect and do not match the results in the tables. The incorrect paragraph read as follows, “High intake of commercial SSBs was reported by 17.3% of adolescents, while 30.0% had moderate intake and 52.7% reported no intake (Supplementary Table 4). Among subgroups, 16.1% of females and 17.9% of males reported high intake. High intake was reported by 16.6% of in-school adolescents and 17.7% of out-of-school adolescents, and by 19.5% of urban adolescents and 14.9% of rural adolescents. Patterns by age and wealth followed broadly similar trends to total SSB consumption while commercial SSB intake was high among adolescents whose mothers had secondary or higher education; detailed estimates are available in Supplementary Table 4.” The correct paragraph should read as, “High intake of traditional SSBs was reported by 38.2% of adolescents, while 42.4% had moderate intake and 19.4% reported low intake (Supplementary Table 4). Among subgroups, high intake of traditional SSBs was reported by 44.9% of out-of-school adolescents and 34.7% of in-school adolescents. High intake was 39.1% among rural adolescents and 37.4% among urban adolescents.”

In **Results**, *SSB Consumption*, paragraph 8, several odds ratios were incorrectly reported. The incorrect paragraph read as follows, “In the adjusted model (Table 5), several factors were associated with greater odds of high vs. moderate traditional SSB intake. Males (OR = 1.13, 95% CI: 1.01–1.27), older adolescents aged 13–16 years (OR = 1.60, 95% CI: 1.37–1.86), out-of-school adolescents (OR = 1.61, 95% CI: 1.29–2.00), and rural residents (OR = 1.14, 95% CI: 1.01–1.28) all had higher odds compared with their respective reference groups. Maternal higher education was protective, with lower odds of high vs. low intake (OR = 0.46, 95% CI: 0.36–0.58). Regional variation was marked: compared with Khyber Pakhtunkhwa, odds were higher in Balochistan (OR = 1.82, 95% CI: 1.25–2.65) and lower in Punjab (OR = 0.74, 95% CI: 0.57–0.96) and Islamabad (OR = 0.60, 95% CI: 0.37–0.99). No significant associations were observed for moderate-to-high vs. low intake”. The correct paragraph with the corrected values should read as, “In the adjusted model (Table 5), several factors were associated with greater odds of high vs. moderate traditional SSB intake. Males (OR = 1.13, 95% CI: 1.01–1.27), older adolescents aged 13–16 years (OR = 1.60, 95% CI: 1.37–1.86), out-of-school adolescents (OR = 2.05, 95% CI: 1.77–2.37), and rural residents (OR = 1.14, 95% CI: 1.01–1.28) all had higher odds compared with their respective reference groups. Maternal higher education was protective, with lower odds of high vs. low intake (OR = 0.46, 95% CI: 0.36–0.58). Regional variation was marked: compared with Khyber Pakhtunkhwa, odds were lower in Balochistan (OR = 0.28, 95% CI: 0.22–0.35), Punjab (OR = 0.26, 95% CI: 0.22–0.30) and Islamabad (OR = 0.20, 95% CI: 0.15–0.27).”

The original version of this article has been updated.

