# Supplementary material for: Consumption of commercial and traditional sugar-sweetened beverages among adolescents in Pakistan: evidence from a national survey
Source: Front Nutr. 2025 Nov 26;12:1679917. doi: 10.3389/fnut.2025.1679917 (PMC12689298; doi:10.3389/fnut.2025.1679917)

**Section 1: Supplementary tables**

Table S1: **Traditional** Sugar-Sweetened Beverage (SSB) Consumption per Week Among Adolescents in Pakistan, 2023 & 2024 by Socio-Demographic Factors (number of servings: Zero or Once, Two to Seven and More than Seven). **Each column represents the odds of moving from the lowest category to a higher category/ category.**

|  | **Zero or One** | **Two to Seven** | **More than 7** | **Total** | **p-value†** |
| --- | --- | --- | --- | --- | --- |
| **Total** | 2754 (19.4) | 6040 (42.4) | 5437 (38.2) | 14232 (100) | <0.0001 |
|  |  |  |  |  |  |
| **Sex, N (%)** |  |  |  |  |  |
| Female | 977 (21.4) | 2022 (44.4) | 1560 (34.2) | 4560 (100) | <0.0001 |
| Male | 1777 (18.4) | 4018 (41.5) | 3877 (40.1) | 9672 (100) |  |
| **Age (Mean, SD)** | 13.42 (1.71) | 13.44 (1.75) | 13.58 (1.81) | 13.48 (1.77) | 0.974 |
| **Age groups (years), N (%)** |  |  |  |  |  |
| 10-12 years old | 835 (20.8) | 1839 (45.8) | 1342 (33.4) | 4017 (100) | 0.053 |
| 13-16 years old | 1919 (18.8) | 4201 (41.1) | 4095 (40.1) | 10215 (100) |  |
| **School status, N (%)** |  |  |  |  |  |
| School going | 1873 (20.0) | 4232 (45.3) | 3243 (34.7) | 9348 (100) | <0.0001 |
| Out-of-school | 881 (18.0) | 1808 (37.0) | 2195 (44.9) | 4884 (100) |  |
| **Wealth Index, N (%)** |  |  |  |  |  |
| Low | 688 (18.5) | 1464 (39.3) | 1574 (42.2) | 3726 (100) | <0.0001 |
| Middle | 839 (17.7) | 2015 (42.6) | 1881 (39.7) | 4734 (100) |  |
| High | 1228 (21.3) | 2561 (44.4) | 1983 (34.4) | 5772 (100) |  |
| **Female caregiver education, N (%)** |  |  |  |  |  |
| No education | 1143 (16.1) | 2792 (39.3) | 3170 (44.6) | 7105 (100) | <0.0001 |
| Primary | 362 (15.7) | 1007 (43.8) | 932 (40.5) | 2301 (100) |  |
| Secondary | 594 (21.7) | 1303 (47.7) | 837 (30.6) | 2734 (100) |  |
| Higher education | 567 (37.9) | 618 (41.3) | 312 (20.9) | 1497 (100) |  |
| **Male caregiver education, N (%)** |  |  |  |  |  |
| No education | 745 (18.2) | 1602 (39.1) | 1751 (42.7) | 4098 (100) | <0.0001 |
| Primary | 432 (18.1) | 1005 (42.2) | 948 (39.7) | 2384 (100) |  |
| Secondary | 749 (16.9) | 2024 (45.5) | 1672 (37.6) | 4445 (100) |  |
| Higher education | 745 (27.1) | 1133 (41.2) | 870 (31.7) | 2747 (100) |  |
| **Parents' occupation status, N (%)** |  |  |  |  |  |
| None of them | 66 (16.0) | 202 (48.9) | 145 (35.1) | 413 (100) | <0.0001 |
| Father only | 2262 (18.9) | 4992 (41.8) | 4692 (39.3) | 11946 (100) |  |
| Mother only | 123 (27.0) | 187 (41.0) | 146 (32.0) | 457 (100) |  |
| Both | 264 (19.8) | 638 (47.9) | 429 (32.2) | 1331 (100) |  |
| **Resident area, N (%)** |  |  |  |  |  |
| Urban | 1597 (21.3) | 3090 (41.3) | 2797 (37.4) | 7484 (100) | 0.064 |
| Rural | 1158 (17.2) | 2950 (43.7) | 2640 (39.1) | 6748 (100) |  |
| **Province, N (%)** |  |  |  |  |  |
| KPK | 238 (7.7) | 1100 (35.7) | 1745 (56.6) | 3084 (100) | <0.0001 |
| Punjab | 1292 (24.9) | 2527 (48.7) | 1372 (26.4) | 5192 (100) |  |
| Sindh | 1044 (20.5) | 2022 (39.6) | 2036 (39.9) | 5103 (100) |  |
| Baluchistan | 173 (20.8) | 381 (45.7) | 280 (33.6) | 834 (100) |  |
| Islamabad | 6 (32.0) | 10 (50.4) | 3 (17.5) | 20 (100) |  |

†P-values from χ² tests for categorical variables and ANOVA for age.

Note: the frequencies (Ns) in the table have been weighted using sampling weights.

Table S2. Associations Between Adolescent’s **Traditional SSB Consumption** (number of servings: Zero or One, Two to Seven and More than Seven) per Week and Socio-Demographic variables among Adolescents in Pakistan, 2023 & 2024 using Generalised Ordinal Logistic Regression Model. **Each column represents the odds of moving from the lowest category to a higher category/ category.**

|  | **Zero or One vs. ≥ Two** | **Two to Seven vs. > Seven** |
| --- | --- | --- |
|  | **(N=13547)** | **(N=13547)** |
|  | **OR (95% CI)** | **OR (95% CI)** |
| **Sex** |  |  |
| Female | 1.00 | 1.00 |
| Male | 1.13 (1.01, 1.27) | 1.13 (1.01, 1.27) |
| **Age groups (years)** |  |  |
| 10-12 years old | 1.00 | 1.00 |
| 13-16 years old | 1.11 (0.94, 1.31) | 1.6 (1.37, 1.86) |
| **School status** |  |  |
| School going | 1.00 | 1.00 |
| Out-of-school | 1.05 (0.88, 1.24) | 2.05 (1.77, 2.37) |
| **Wealth Index** |  |  |
| Low | 1.00 | 1.00 |
| Middle | 1.09 (0.94, 1.26) | 1.09 (0.94, 1.26) |
| High | 1.02 (0.87, 1.19) | 1.02 (0.87, 1.19) |
| **Female caregiver education** |  |  |
| No education | 1.00 | 1.00 |
| Primary | 0.93 (0.79, 1.08) | 0.93 (0.79, 1.08) |
| Secondary | 0.63 (0.53, 0.74) | 0.63 (0.53, 0.74) |
| Higher education | 0.33 (0.26, 0.41) | 0.46 (0.36, 0.58) |
| **Male caregiver education** |  |  |
| No education | 1.00 | 1.00 |
| Primary | 1.05 (0.88, 1.25) | 1.05 (0.88, 1.25) |
| Secondary | 1.5 (1.24, 1.81) | 1.21 (1.01, 1.43) |
| Higher education | 1.3 (1.07, 1.57) | 1.3 (1.07, 1.57) |
| **Parents' occupation status** |  |  |
| None of them | 1.00 | 1.00 |
| Father only | 1.03 (0.72, 1.47) | 1.03 (0.72, 1.47) |
| Mother only | 0.83 (0.49, 1.43) | 0.83 (0.49, 1.43) |
| Both | 1.42 (0.95, 2.14) | 0.93 (0.63, 1.38) |
| **Resident area** |  |  |
| Urban | 1.00 | 1.00 |
| Rural | 1.14 (1.01, 1.28) | 1.14 (1.01, 1.28) |
| **Province** |  |  |
| KPD | 1.00 | 1.00 |
| Punjab | 0.26 (0.22, 0.30) | 0.26 (0.22, 0.30) |
| Sindh | 0.33 (0.27, 0.39) | 0.59 (0.50, 0.69) |
| Baluchistan | 0.28 (0.22, 0.35) | 0.28 (0.22, 0.35) |
| Islamabad | 0.20 (0.15, 0.27) | 0.20 (0.15, 0.27) |

Note: the model included sampling weights.

**SEPARATE TABLES OF TABLE 1 (IN THE MAIN TABLES) FOR SEX AND SCHOOL STATUS.**

Table S3. Characteristics of the study sample (total and by Sex) among adolescents in Pakistan, 2023 & 2024

|  | **Female** | **Male** | **Overall** |
| --- | --- | --- | --- |
| **Total** | 5221 (100) | 9011 (100) | 14232 (100) |
|  |  |  |  |
| **School status, N (%)** |  |  |  |
| School going | 3448 (67.3) | 5563 (61.1) | 9011 (63.3) |
| Out-of-School | 1678 (32.7) | 3543 (38.9) | 5221 (36.7) |
| **Age (Mean, SD)** | 13.34 (1.76) | 13.57 (1.76) | 13.48 (1.77) |
| **Age groups (years), N (%)** |  |  |  |
| 10-12 | 1665 (32.5) | 2595 (28.5) | 4260 (29.9) |
| 13-16 | 3461 (67.5) | 6511 (71.5) | 9972 (70.1) |
| **Wealth Index, N (%)** |  |  |  |
| Low | 1544 (30.1) | 3283 (36.1) | 4827 (33.9) |
| Middle | 1732 (33.8) | 3142 (34.5) | 4874 (34.3) |
| High | 1850 (36.1) | 2681 (29.4) | 4531 (31.8) |
| **Female caregiver education, N (%)** |  |  |  |
| No education | 2733 (53.3) | 5426 (59.6) | 8159 (57.3) |
| Primary | 833 (16.3) | 1253 (13.8) | 2086 (14.7) |
| Secondary | 911 (17.8) | 1272 (14) | 2183 (15.3) |
| Higher education | 545 (10.6) | 769 (8.4) | 1314 (9.2) |
| **Male caregiver education, N (%)** |  |  |  |
| No education | 1689 (33.0) | 3328 (36.6) | 5017 (35.3) |
| Primary | 760 (14.8) | 1552 (17) | 2312 (16.3) |
| Secondary | 1523 (29.7) | 2339 (25.7) | 3862 (27.1) |
| Higher education | 1019 (19.9) | 1548 (17) | 2567 (18.0) |
| **Parents' occupation status, N (%)** |  |  |  |
| None of them | 96 (1.9) | 280 (3.1) | 376 (2.6) |
| Father only | 4090 (79.8) | 7379 (81) | 11469 (80.6) |
| Mother only | 238 (4.6) | 300 (3.3) | 538 (3.8) |
| Both | 676 (13.2) | 1074 (11.8) | 1750 (12.3) |
| **Resident area, N (%)** |  |  |  |
| Urban | 2189 (42.7) | 4261 (46.8) | 6450 (45.3) |
| Rural | 2937 (57.3) | 4845 (53.2) | 7782 (54.7) |
| **Province, N (%)** |  |  |  |
| KPK | 781 (15.2) | 1712 (18.8) | 2493 (17.5) |
| Punjab | 2056 (40.1) | 2979 (32.7) | 5035 (35.4) |
| Sindh | 1286 (25.1) | 2747 (30.2) | 4033 (28.3) |
| Baluchistan | 846 (16.5) | 1211 (13.3) | 2057 (14.5) |
| Islamabad | 157 (3.1) | 457 (5.0) | 614 (4.3) |
| **Total SSB consumption per week, N (%)** |  |  |  |
| Zero | 233 (4.6) | 305 (3.4) | 538 (3.8) |
| One | 252 (4.9) | 249 (2.7) | 501 (3.5) |
| Two-Four | 310 (6.1) | 599 (6.6) | 909 (6.4) |
| Five-Six | 46 (0.9) | 76 (0.8) | 122 (0.7) |
| Seven | 902 (17.6) | 1229 (13.5) | 2131 (15.0) |
| More than seven | 3383 (66.0) | 6648 (73.0) | 10031 (70.5) |
| **Commercial SSB consumption per week, N (%)** |  |  |  |
| Zero | 1883 (36.7) | 2874 (31.6) | 4757 (33.4) |
| One | 981 (19.1) | 1531 (16.8) | 2512 (17.7) |
| Two-Four | 610 (11.9) | 1748 (19.2) | 2358 (16.6) |
| Five-Six | 182 (3.6) | 375 (4.1) | 557 (3.9) |
| Seven | 261 (5.1) | 500 (5.5) | 761 (5.4) |
| More than seven | 1209 (23.6) | 2078 (22.8) | 3287 (23.1) |
| **Traditional consumption per week, N (%)** |  |  |  |
| Zero | 487 (9.5) | 701 (7.7) | 1188 (8.4) |
| One | 619 (12.1) | 988 (10.9) | 1607 (11.3) |
| Two-Four | 208 (4.1) | 251 (2.8) | 459 (3.2) |
| Five-Six | 216 (4.2) | 436 (4.8) | 652 (4.6) |
| Seven | 1963 (38.3) | 3189 (35) | 5152 (36.2) |
| More than seven | 1633 (31.9) | 3541 (38.9) | 5174 (36.4) |

Note: the frequencies (Ns) in the table are unweighted.

Table S4. Characteristics of the study sample (total and by school status) among adolescents in Pakistan, 2023 & 2024

|  | **Out-of-School** | **School-going** | **Overall** |
| --- | --- | --- | --- |
| **Total** | 5221 (100) | 9011 (100) | 14232 (100) |
|  |  |  |  |
| **Sex, N (%)** |  |  |  |
| Female | 1678 (32.1) | 3448 (38.3) | 5126 (36.0) |
| Male | 3543 (67.9) | 5563 (61.7) | **9106 (64.0)** |
| **Age (Mean, SD)** | 12.82 (1.92) | 13.87 (1.54) | 13.48 (1.77) |
| **Age groups (years), N (%)** |  |  |  |
| 10-12 | 2403 (46.0) | 1857 (20.6) | 4260 (29.9) |
| 13-16 | 2818 (54.0) | 7154 (79.4) | 9972 (70.1) |
| **Wealth Index, N (%)** |  |  |  |
| Low | 2729 (52.3) | 2098 (23.3) | 4827 (33.9) |
| Middle | 1592 (30.5) | 3282 (36.4) | 4874 (34.3) |
| High | 900 (17.2) | 3631 (40.3) | 4531 (31.8) |
| **Female caregiver education, N (%)** |  |  |  |
| No education | 3978 (76.2) | 4181 (46.4) | 8159 (57.3) |
| Primary | 609 (11.7) | 1477 (16.4) | 2086 (14.7) |
| Secondary | 351 (6.7) | 1832 (20.3) | 2183 (15.3) |
| Higher education | 86 (1.7) | 1228 (13.6) | 1314 (9.2) |
| **Male caregiver education, N (%)** |  |  |  |
| No education | 2734 (52.4) | 2283 (25.3) | 5017 (35.3) |
| Primary | 1005 (19.3) | 1307 (14.5) | 2312 (16.3) |
| Secondary | 947 (18.1) | 2915 (32.4) | 3862 (27.1) |
| Higher education | 350 (6.7) | 2217 (24.6) | 2567 (18.0) |
| **Parents' occupation status, N (%)** |  |  |  |
| None of them | 166 (3.2) | 210 (2.3) | 376 (2.6) |
| Father only | 4016 (76.9) | 7453 (82.7) | 11469 (80.6) |
| Mother only | 217 (4.2) | 321 (3.6) | 538 (3.8) |
| Both | 798 (15.3) | 952 (10.6) | 1750 (12.3) |
| **Resident area, N (%)** |  |  |  |
| Urban | 2145 (41.1) | 4305 (47.8) | 6450 (45.3) |
| Rural | 3076 (58.9) | 4706 (52.2) | 7782 (54.7) |
| **Province, N (%)** |  |  |  |
| KPK | 893 (17.1) | 1600 (17.8) | 2493 (17.5) |
| Punjab | 1781 (34.1) | 3254 (36.1) | 5035 (35.4) |
| Sindh | 1429 (27.4) | 2604 (28.9) | 4033 (28.3) |
| Baluchistan | 905 (17.3) | 1152 (12.8) | 2057 (14.5) |
| Islamabad | 213 (4.1) | 401 (4.5) | 614 (4.3) |
| **Total SSB consumption per week, N (%)** |  |  |  |
| Zero | 172 (3.3) | 366 (4.1) | 538 (3.8) |
| One | 134 (2.6) | 367 (4.1) | 501 (3.5) |
| Two-Four | 281 (5.4) | 628 (7.0) | 909 (6.4) |
| Five-Six | 68 (1.3) | 54 (0.6) | 122 (0.9) |
| Seven | 775 (14.8) | 1356 (15.1) | 2131 (15.0) |
| More than seven | 3791 (72.6) | 6240 (69.3) | 10031 (70.5) |
| **Commercial SSB consumption per week, N (%)** |  |  |  |
| Zero | 2009 (38.5) | 2748 (30.5) | 4757 (33.4) |
| One | 817 (15.7) | 1695 (18.8) | 2512 (17.7) |
| Two-Four | 891 (17.1) | 1467 (16.3) | 2358 (16.6) |
| Five-Six | 265 (5.1) | 292 (3.2) | 557 (3.9) |
| Seven | 185 (3.5) | 576 (6.4) | 761 (5.4) |
| More than seven | 1054 (20.2) | 2233 (24.8) | 3287 (23.1) |
| **Traditional consumption per week, N (%)** |  |  |  |
| Zero | 368 (7.1) | 820 (9.1) | 1188 (8.4) |
| One | 446 (8.5) | 1161 (12.9) | 1607 (11.3) |
| Two-Four | 181 (3.5) | 278 (3.1) | 459 (3.2) |
| Five-Six | 282 (5.4) | 370 (4.1) | 652 (4.6) |
| Seven | 1613 (30.9) | 3539 (39.3) | 5152 (36.2) |
| More than seven | 2331 (44.7) | 2843 (31.6) | 5174 (36.4) |

Note: the frequencies (Ns) in the table are unweighted.

Table S5. Commercial Sugar-Sweetened Beverage (SSB) Consumption per Week Among Adolescents in Pakistan, 2023 & 2024 by Socio-Demographic Factors.

|  | **Zero or One** | **Two to Seven** | **More than 7** | **Total** | **p-value**† |
| --- | --- | --- | --- | --- | --- |
| **Total, N (%)** | 7505 (52.7) | 4264 (30.0) | 2463 (17.3) | 14232 (100) | <0.0001 |
|  |  |  |  |  |  |
| **Sex, N (%)** |  |  |  |  |  |
| Female | 2646 (58.0) | 1179 (25.9) | 735 (16.1) | 4560 (100) | <0.0001 |
| Male | 4859 (50.2) | 3085 (31.9) | **1728 (17.9)** | 9672 (100) |  |
| **Age (Mean, SD)** | 13.3 (1.82) | 13.65 (1.76) | 13.71 (1.61) | 13.48 (1.77) | <0.0001 |
| **Age groups (years), N (%)** |  |  |  |  |  |
| 10-12 years old | 2365 (58.9) | 1058 (26.3) | 593 (14.8) | 4017 (100) | <0.0001 |
| 13-16 years old | 5140 (50.3) | 3206 (31.4) | **1869 (18.3)** | 10215 (100) |  |
| **School status, N (%)** |  |  |  |  |  |
| School-going | 2604 (53.3) | 1468 (30.1) | 813 (16.6) | 4884 (100) | <0.0001 |
| Out-of-School | 4902 (52.4) | 2796 (29.9) | **1650 (17.7)** | 9348 (100) |  |
| **Wealth Index, N (%)** |  |  |  |  |  |
| Low | 2178 (58.5) | 1008 (27.1) | 540 (14.5) | 3726 (100) | <0.0001 |
| Middle | 2270 (48.0) | 1469 (31.0) | **995 (21.0)** | 4734 (100) |  |
| High | 3057 (53.0) | 1786 (31.0) | 929 (16.1) | 5772 (100) |  |
| **Female caregiver education, N (%)** |  |  |  |  |  |
| No education | 4026 (56.7) | 1969 (27.7) | 1110 (15.6) | 7105 (100) | <0.0001 |
| Primary | 1261 (54.8) | 695 (30.2) | 345 (15.0) | 2301 (100) |  |
| Secondary | 1226 (44.9) | 884 (32.3) | **624 (22.8)** | 2734 (100) |  |
| Higher education | 684 (45.7) | 504 (33.7) | **309 (20.6)** | 1497 (100) |  |
| **Male caregiver education, N (%)** |  |  |  |  |  |
| No education | 2410 (58.8) | 1119 (27.3) | 569 (13.9) | 4098 (100) | <0.0001 |
| Primary | 1255 (52.6) | 712 (29.9) | 417 (17.5) | 2384 (100) |  |
| Secondary | 2314 (52.1) | 1296 (29.2) | 835 (18.8) | 4445 (100) |  |
| Higher education | 1240 (45.1) | 947 (34.5) | **560 (20.4)** | 2747 (100) |  |
| **Parents' occupation status, N (%)** |  |  |  |  |  |
| None of them | 252 (60.9) | 111 (26.9) | 51 (12.2) | 413 (100) | <0.0001 |
| Father only | 6381 (53.4) | 3619 (30.3) | 1947 (16.3) | 11946 (100) |  |
| Mother only | 215 (47.1) | 100 (21.8) | **142 (31.0)** | 457 (100) |  |
| Both | 619 (46.5) | 413 (31.0) | 299 (22.5) | 1331 (100) |  |
| **Resident area, N (%)** |  |  |  |  |  |
| Urban | 3342 (44.7) | 2686 (35.9) | **1455 (19.5)** | 7484 (100) | <0.0001 |
| Rural | 4163 (61.7) | 1578 (23.4) | 1007 (14.9) | 6748 (100) |  |
| **Province, N (%)** |  |  |  |  |  |
| KPK | 1888 (61.2) | 886 (28.7) | 310 (10.1) | 3084 (100) | <0.0001 |
| Punjab | 3490 (67.2) | 1234 (23.8) | 467 (9.0) | 5192 (100) |  |
| Sindh | 2023 (39.6) | 1999 (39.2) | 1081 (21.2) | 5103 (100) |  |
| Baluchistan | 91 (11.0) | 141 (16.9) | **602 (72.1)** | 834 (100) |  |
| Islamabad | 13 (68.0) | 4 (19.5) | 2 (12.5) | 20 (100) |  |

†P-values from χ² tests for categorical variables and ANOVA for age.

Note: the frequencies (Ns) in the table have been weighted using sampling weights.

Table S6: Traditional Sugar-Sweetened Beverage (SSB) Consumption per Week Among Adolescents in Pakistan, 2023 & 2024 by Socio-Demographic Factors (number of servings: Zero or Once, Two to Seven and More than Seven). Each column represents the odds of moving from the lowest category to a higher category/ category.

|  | **Zero or One** | **Two to Seven** | **More than 7** | **Total** | **p-value†** |
| --- | --- | --- | --- | --- | --- |
| **Total** | 2754 (19.4) | 6040 (42.4) | 5437 (38.2) | 14232 (100) | <0.0001 |
|  |  |  |  |  |  |
| **Sex, N (%)** |  |  |  |  |  |
| Female | 977 (21.4) | 2022 (44.4) | 1560 (34.2) | 4560 (100) | <0.0001 |
| Male | 1777 (18.4) | 4018 (41.5) | 3877 (40.1) | 9672 (100) |  |
| **Age (Mean, SD)** | 13.42 (1.71) | 13.44 (1.75) | 13.58 (1.81) | 13.48 (1.77) | 0.974 |
| **Age groups (years), N (%)** |  |  |  |  |  |
| 10-12 years old | 835 (20.8) | 1839 (45.8) | 1342 (33.4) | 4017 (100) | 0.053 |
| 13-16 years old | 1919 (18.8) | 4201 (41.1) | 4095 (40.1) | 10215 (100) |  |
| **School status, N (%)** |  |  |  |  |  |
| School going | 1873 (20.0) | 4232 (45.3) | 3243 (34.7) | 9348 (100) | <0.0001 |
| Out-of-school | 881 (18.0) | 1808 (37.0) | 2195 (44.9) | 4884 (100) |  |
| **Wealth Index, N (%)** |  |  |  |  |  |
| Low | 688 (18.5) | 1464 (39.3) | 1574 (42.2) | 3726 (100) | <0.0001 |
| Middle | 839 (17.7) | 2015 (42.6) | 1881 (39.7) | 4734 (100) |  |
| High | 1228 (21.3) | 2561 (44.4) | 1983 (34.4) | 5772 (100) |  |
| **Female caregiver education, N (%)** |  |  |  |  |  |
| No education | 1143 (16.1) | 2792 (39.3) | 3170 (44.6) | 7105 (100) | <0.0001 |
| Primary | 362 (15.7) | 1007 (43.8) | 932 (40.5) | 2301 (100) |  |
| Secondary | 594 (21.7) | 1303 (47.7) | 837 (30.6) | 2734 (100) |  |
| Higher education | 567 (37.9) | 618 (41.3) | 312 (20.9) | 1497 (100) |  |
| **Male caregiver education, N (%)** |  |  |  |  |  |
| No education | 745 (18.2) | 1602 (39.1) | 1751 (42.7) | 4098 (100) | <0.0001 |
| Primary | 432 (18.1) | 1005 (42.2) | 948 (39.7) | 2384 (100) |  |
| Secondary | 749 (16.9) | 2024 (45.5) | 1672 (37.6) | 4445 (100) |  |
| Higher education | 745 (27.1) | 1133 (41.2) | 870 (31.7) | 2747 (100) |  |
| **Parents' occupation status, N (%)** |  |  |  |  |  |
| None of them | 66 (16.0) | 202 (48.9) | 145 (35.1) | 413 (100) | <0.0001 |
| Father only | 2262 (18.9) | 4992 (41.8) | 4692 (39.3) | 11946 (100) |  |
| Mother only | 123 (27.0) | 187 (41.0) | 146 (32.0) | 457 (100) |  |
| Both | 264 (19.8) | 638 (47.9) | 429 (32.2) | 1331 (100) |  |
| **Resident area, N (%)** |  |  |  |  |  |
| Urban | 1597 (21.3) | 3090 (41.3) | 2797 (37.4) | 7484 (100) | 0.064 |
| Rural | 1158 (17.2) | 2950 (43.7) | 2640 (39.1) | 6748 (100) |  |
| **Province, N (%)** |  |  |  |  |  |
| KPK | 238 (7.7) | 1100 (35.7) | 1745 (56.6) | 3084 (100) | <0.0001 |
| Punjab | 1292 (24.9) | 2527 (48.7) | 1372 (26.4) | 5192 (100) |  |
| Sindh | 1044 (20.5) | 2022 (39.6) | 2036 (39.9) | 5103 (100) |  |
| Baluchistan | 173 (20.8) | 381 (45.7) | 280 (33.6) | 834 (100) |  |
| Islamabad | 6 (32.0) | 10 (50.4) | 3 (17.5) | 20 (100) |  |

†P-values from χ² tests for categorical variables and ANOVA for age.

Note: the frequencies (Ns) in the table have been weighted using sampling weights.

**Section 2.** To address the covariates violating Proportional Odds (PO) assumption in the dataset, Generalized Ordinal Logistic Regression Model (a Partial Proportional Odds (PPO) model) was used to accommodate the effect. As the PO assumption was violated, Parents' occupation status does not have a uniform effect across all levels of SSB consumption.

For example, in Table 4, Father-only occupation status has 2.06 times higher odds than neither parents’ occupation status of drinking more SSBs, but the strength of this relationship varies depending on the threshold. The effect of being Father only occupation status is stronger (OR = 2.06) at the lower threshold (moving from Zero or One to Two or more SSBs per week) than at the higher threshold (moving from Two to Seven to More than Seven SSBs per week, (OR = 1.30) and not statistically significant). This suggests that farther only occupation status has a stronger influence on the likelihood of initiating moderate SSB consumption than on the likelihood of moving from moderate to excessive consumption.

| **Variable** | **Level** | **P-value** |
| --- | --- | --- |
| Parents' occupation status | 1. Father only (3) Both | 0.02785 and 0.01219 |
| Province | (5) Islamabad | 0.00417 |

**Section 3. Assessing Frequency of Sugar-Sweetened Beverage (SSB) Consumption and Construction of the Wealth Index**

**2.1. SSB Calculation method**

To estimate SSB we used a subset of questions of a food frequency questionnaire (FFQ) validated for adolescents [^22^](https://paperpile.com/c/oFnOlk/8QHQ). SSBs were classified into four categories according to previous studies done in South Asia as [^23^](https://paperpile.com/c/oFnOlk/6n6p): 1) **Soft carbonated drinks**, defined as any carbonated beverage containing added sugars (e.g., Coke, Fanta, Sprite); 2) **Fruit Drinks**, which include beverages with added sugar but exclude 100% fruit juice [^24^](https://paperpile.com/c/oFnOlk/M42f); 3) **Sweetened traditional drinks**, referring to tea-, yogurt-, milk-, and coffee-based drinks with added sugars; and 4) **Energy Drinks**, characterised by their sugar content alongside energy-boosting ingredients[^23^](https://paperpile.com/c/oFnOlk/6n6p).

Participants were asked to report their consumption of SSBs over the past seven days using the question: “During the past seven days, how many times did you drink [specific beverage category]?” Response options included: (1) Never, (2) 1 time per week, (3) 2-4 times per week, (4) 5-6 times per week, (5) Once a day, every day, and (6) Every day, more than once. Participants who selected the last option, (6), were asked a follow-up question: “How many times per day?”.

Based on participants' responses, we calculated the total weekly average frequency of consumption for each SSB category. The following values were assigned to each response category: Never = 0 times per week; 1 time per week = 1; 2-4 times per week = 3 (mean); 5-6 times per week = 5.5 (mean); and once daily = 7. For participants who reported consuming SSBs more than once per day, the weekly frequency was calculated based on their reported daily intake. In cases where participants who consumed SSBs more than once per day could not recall the exact number of daily consumptions, we assigned a conservative minimum estimate of 14 times per week (i.e., twice daily).

Example calculation:

Question:

During the past seven days, how many times did you drink (either a glass, can or bottle) Coke, Pepsi, Sprite, 7-Up, Fanta, Mountain Dew or other soft carbonated drinks?

1. Never

2. Once a day, one day in a week

3. Once a day, every day

4. Every day, more than once.

5. One time 2–4 days a week

6. One time 5-6 days a week

7. Every day

 If 7 is selected ask how many times a day ___________

Table S5. Number of servings assigned based on responses to the questions regarding past seven days' SSB intake per week among adolescents in Pakistan, 2023 & 2024 .

| **Responses**  **(in the past 7 days)** | **Frequency per week** |
| --- | --- |
| 1. Never | 0 |
| 1. 1 time per week | 1 |
| 1. 2-4 times per week | 3 |
| 1. 5-6 times per week | 5.5 |
| 1. Once a day, every day | 7 |
| 1. Every day more than once^†^ | 14 |

^†^We recorded the number of drinks consumed per day as reported by each adolescent. For adolescents who couldn't recall the exact frequency, we assigned the minimum value of 14 drinks per week to these cases.

The total weekly average frequency of consumption for each SSB category—soft carbonated drinks, fruit drinks, sweetened traditional drinks, and energy drinks—was summed for each participant to calculate their total SSB consumption per week.

For example, if an adolescent reported consuming 2 servings of soft carbonated drinks, 5 of fruit drinks, 3 of sweetened traditional drinks, and 2 of energy drinks, then the total SSB consumption score would be 12 (2 + 5 + 3 + 2). The total Commercial SSB consumption score (soft carbonated drinks, fruit drinks, and energy drinks) would be 9, and the total traditional SSB consumption score (sweetened traditional drinks) would be 3.

**2.2. Wealth Index**

The analysis of wealth quintiles involved constructing a composite wealth index based on key household asset variables, including flush toilet, television, refrigerator, car, and moped/scooter/motorcycle. Variables such as electricity, fixed telephone, mobile phone, and radio were excluded because they were owned by either more than 95% or less than 5% of the sample, thus offering limited variability and discriminatory power for assessing wealth. Principal component analysis (PCA) was applied to the selected variables to generate a wealth index that effectively captured socioeconomic differences. This index was then divided into three categories such as low, middle, and high wealth quintiles, providing a stratified representation of the population’s socioeconomic groups for further analysis.


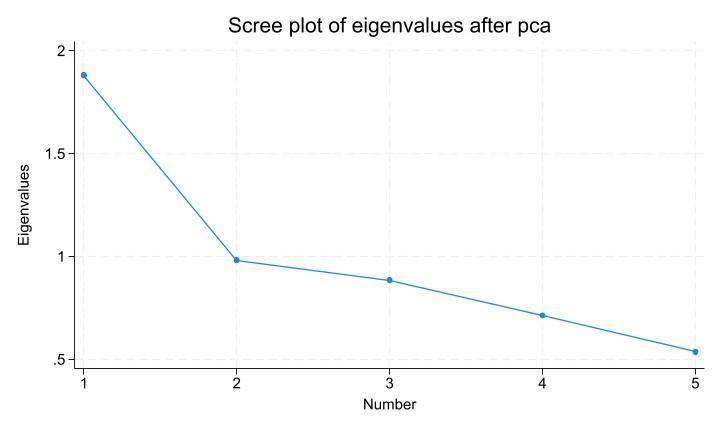

Supplement: Supplementary file 1 [file Supplementary_file_1.docx]
