# Supplementary material for: Consumption of commercial and traditional sugar-sweetened beverages among adolescents in Pakistan: evidence from a national survey
Source: Front Nutr. 2025 Nov 26;12:1679917. doi: 10.3389/fnut.2025.1679917 (PMC12689298; doi:10.3389/fnut.2025.1679917)
Supplement: Supplementary file 2 [file Supplementary_file_2.docx]

**Technical note on preparation of sampling weights of National cross-sectional survey of in-school and out-of-school children in Pakistan**

*Sampling strategy for school children*

Multistage sampling weights developed for the **school-going children.**

**At first stage** district level weights were prepared, as two districts from each province selected using Simple Random Sampling (SRS) and Islamabad selected with certainty.

Probability of selection of districts in a province = P_1_ = number of selected districts in a province / total number of districts in a province

While, first stage weights = W_1_ = 1/ P_1_

**At second stage**; Patwar Circle/ Circles from each district selected using Probability Proportional to Size (PPS) method using estimated number of households in a Patwar Circle / Circle as Measure of Size (MOS) with Systematic Random Sampling (SYS). It is noteworthy that Patwar Circles selected from rural area while Circles selected from urban area.

Probability of Section of Patwar Circle/Circle = P_2_

Where, P_2_ = (Total number of households in a selected Patwar Circles/ Circles as per 2017- Population & Housing Census Sampling Frame) / (Total number of households in all Patwar Circles / Circles of a district as per as per 2017- Population & Housing Census Sampling Frame) * Number of selected Patwar Circles/ Circles

Second Stage weight=1/P_2_=W_2_

**At third stage**, two schools from each Patwar Circle/ Circles selected using Simple Random Sampling (SRS).

Third stage probability of selection of schools = P_3_

Where, P_3 =_ number of selected schools / total number of schools in a Patwar Circle/ Circle

Third stage weight = W_3_= 1/ P_3_

**At fourth stage;** children of 10-16 years of age from 6-10 class interviewed. From each school; 50 interviews completed.

Fourth Stage Probability of selection of children= P_4_

Where, P_4 =_ total number of complete interviews / total number of target interviews

**The overall probability of selection** of children in a sample is

P (School survey) = (P_1 *_ P_2 *_ P_3*_ P_4)_

**The overall weight** of selection of children in a sample is

W_t_ (school survey) = 1/ (P_1 *_ P_2 *_ P_3*_ P_4)_

**Sampling of Out-of-School children**

Multistage sampling weights developed for the **Out-of-School children (household survey).**

**At first stage:** In pre-selected Patwar Circle/ Circles, certain number of Patwar Circle/ Circles selected using Simple Random Sampling (SRS).

**At second stage:** Later on, 1-3 enumeration blocks selected from each Patwar Circle/ Circles using Simple Random Sampling (SRS).

**At third stage**: In selected enumeration blocks, the households having Out of School Children selected for enumeration using Systematic Random Sampling technique (SYS).

Therefore, the **probability of selection of Patwar Circle/ Circle** in pre-selected Patwar Circles/ Circles = P_5_

Where, P_5_ = (number of selected Patwar Circle/ Circle in a district) / (Total number of pre-selected Patwar Circle/ Circle in a district)

Weight of selection of enumeration blocks= W_5_ = 1/ P_5_

The **probability of selection of enumeration blocks** in selected Patwar Circles/ Circles = P_6_

Where, P_6_ = (number of selected enumeration blocks in a Patwar Circle/ Circle) / (Total number of enumeration blocks in a Patwar Circle/ Circle as per 2017- Population & Housing Census Sampling Frame)

Weight of selection of enumeration blocks= W_6_ = 1/ P_6_

In selected enumeration block, OOSC household enumerated. The **probability of selection of OOSC** household = P_7_

Where, P_7_ = Total number of completed OOSC interviews / Total number of target OOSC interviews

The **overall probability of selection of OOSC** in sample

P( household Survey) = (P_1 *_ P_2 *_ P_5 *_ P_6 *_ P_7 )_

The **overall weight of selection of OOSC** in a sample = W_t_ (household survey) = 1/ (P_1 *_ P_2 *_ P_5 *_ P_6 *_ P_7)_
